# Clinical Characterization and Possible Pathological Mechanism of Acute Myocardial Injury in COVID-19

**DOI:** 10.3389/fcvm.2022.862571

**Published:** 2022-03-21

**Authors:** Siyi Li, Jinan Wang, Yan Yan, Zekun Zhang, Wei Gong, Shaoping Nie

**Affiliations:** ^1^Coronary Heart Disease Center, Department of Cardiology, Beijing Anzhen Hospital, Capital Medical University, Beijing, China; ^2^Beijing Institute of Heart, Lung, and Blood Vessel Diseases, Beijing, China; ^3^The Affiliated Rehabilitation Hospital of Chongqing Medical University, Chongqing, China

**Keywords:** COVID-19, myocardial injury, inflammation markers, angiotensin-converting enzyme-2 receptor, prognosis

## Abstract

COVID-19 is a respiratory disease that can cause damage to multiple organs throughout the body. Cardiovascular complications related to COVID-19 mainly include acute myocardial injury, heart failure, acute coronary syndrome, arrhythmia, myocarditis. Among them, myocardial injury is the most common complication in COVID-19 hospitalized patients, and is associated with poor prognosis such as death and arrhythmias. There is a continuous relationship between myocardial injury and the severity of COVID-19. The incidence of myocardial injury is higher in critically ill patients and dead patients, and myocardial injury is more likely to occur in the elderly critically ill patients with comorbidities. Myocardial injury is usually accompanied by more electrocardiogram abnormalities, higher inflammation markers and more obvious echocardiographic abnormalities. According to reports, COVID-19 patients with a history of cardiovascular disease have a higher in-hospital mortality, especially in the elder patients. At present, the mechanism of myocardial injury in COVID-19 is still unclear. There may be direct injury of myocardial cells, systemic inflammatory response, hypoxia, prethrombotic and procoagulant state, myocardial interstitial fibrosis, interferon-mediated immune response and coronary artery plaque instability and other related factors, and angiotensin-converting enzyme-2 receptor may play a key role in the myocardial injury in COVID-19.

## Introduction

Coronavirus disease 2019 (COVID-19), a severe acute respiratory infectious disease caused by severe acute respiratory syndrome coronavirus-2 (SARS-CoV-2), has caused widespread epidemics worldwide since its outbreak in 2019. According to real-time data, as of February 2022, the cumulative number of confirmed cases of COVID-19 worldwide exceeded 393 million, and the number of deaths exceeded 5 million, especially among the elderly and those with other complications such as diabetes, hypertension, and cardiovascular disease (1). The current epidemic continues to exist and is in a recurring stage. Although the use rate of vaccines is getting higher and higher, the number of infections continues to rise.

COVID-19 mainly affects the respiratory system. The most common early symptoms are fever (80–90%), dry cough (60–70%) and shortness of breath (53–80%) (2). With the increase in the number of infected people, more and more clinical evidence shows that COVID-19 can damage various systems of the human body to varying degrees, and the damage to the cardiovascular system is particularly serious. Previous literature found that cardiovascular manifestations related to COVID-19 include acute myocardial injury, myocarditis, cardiomyopathy, acute coronary syndrome and myocardial infarction, arrhythmia, cardiac arrest, cardiac tamponade, Kawasaki disease, cardiovascular thromboembolism (including venous thromboembolism, stroke) and so on (3–8). In addition, doctors from many countries have reported that compared with the general population, patients with cardiovascular disease have a worse prognosis for SARS-CoV-2 infection. Previous studies suggested that COVID-19 patients with cardiovascular disease are often more severely ill and have a higher risk of death, especially in elderly patients (9). It can be seen that COVID-19 and cardiovascular diseases affect each other and together lead to acute and malignant adverse events. Among the cardiovascular manifestations related to COVID-19, arrhythmia is the most common, with an incidence rate of up to 44%. The mortality rate of cardiac injury is the highest, about 50% (10). Therefore, it is particularly important to pay attention to the pathological mechanism, clinical manifestations, disease process, and prognosis of heart damage caused by COVID-19.

At present, the mechanisms of cardiac injury caused by COVID-19 are still unclear. Viruses directly damage cardiomyocytes, systemic inflammatory storms, immune response disorders, endothelial injury and thrombotic inflammation, and functional maladaptation of angiotensin-converting enzyme-2 (ACE-2) receptor-related pathways may all contribute to COVID-19's serious damage to myocardium (11). This review focused on the myocardial injury caused by COVID-19 and summarized the relevant clinical features and pathogenesis.

## Clinical Characterization of Myocardial Injury In Covid-19

Acute myocardial injury is the most common extrapulmonary manifestation of COVID-19. The definition of acute myocardial injury in COVID-19 is blood levels of cardiac biomarkers [high-sensitivity troponin I (hs-TnI)] above the 99th percentile upper reference limit. The existing literature reports that the incidence of acute myocardial injury ranges from 12 to 100%. Almost all patients with severe illness have severe myocardial damage, which is close to 100% in critically ill patients and 70 to 80% in severely ill patients. Critically ill patients requiring intensive care unit (ICU) admission are approximately 13 times more likely to have acute cardiac injury than non-critically ill patients (12).

Patients suspected of cardiac injury caused by COVID-19 often experience chest pain alongside other viral systemic symptoms, including fever, cough, and/or dyspnea (13). Patients with acute myocardial injury tend to be older, have more comorbidities and more severe illness. In addition, the symptoms of respiratory system of patients with acute myocardial injury caused by COVID-19 is relatively more severe. Studies showed that 46.3% of patients with acute myocardial injury required non-invasive mechanical ventilation, compared with 3.9% of patients without myocardial injury. Similarly, the demand for invasive mechanical ventilation in patients with and without heart injury increased by 22 and 4.2%, respectively, and the incidence of acute respiratory distress syndrome (ARDS) in patients with myocardial injury also increased significantly (58.5 vs 14.7%, *P* < 0.001) (14).

In COVID-19 patients, cardiovascular markers such as creatine kinase (CK), creatinine, TnI (heart), brain natriuretic peptide (BNP), lactate dehydrogenase (LDH), alanine aminotransferase, aspartate aminotransferase and D-dimer concentration significantly increased, which may indicate the occurrence of acute myocardial injury (15). Guo et al. found that among 187 COVID-19 patients with an average age of 58.5 years, 52 patients suffered from acute myocardial injury, with a mean CK-MB fraction of 3.34 ng/ml; mean myoglobin of 128.7 μg/ml (16). Shi et al. found that among 416 COVID-19 infected patients, 19.7% of the patients had myocardial injury, which was defined as increased hs-cTnI and NT-proBNP (14). In a retrospective cohort study, Zhou et al. found that 33 out of 191 COVID-19 patients (17%) had acute myocardial injury, of which 24 patients showed high-sensitivity cardiac troponin levels higher than 28 pg/ml (4).

In addition, the abnormal results of other laboratory and imaging examination also indicated the occurrence of acute myocardial injury. The 12-lead electrocardiogram showed low voltage, diffuse ST-segment elevation, T-wave inversion, PR segment depression and new Q waves (13, 14), and echocardiogram showed diffuse myocardial dyskinesia and decreased ejection fraction always suggest that myocardial injury occur (13, 17). Besides, cardiovascular magnetic resonance (CMR) revealed 78% German patients who recovered from COVID-19 recently had myocardial involvement. The abnormal CMR findings included increased myocardial native T1 (73%), raised myocardial native T2 (60%), myocardial late gadolinium enhancement (32%), or pericardial enhancement (22%) (18). It was reported in the previous literature that in severe cases, direct acute myocardial injury caused by COVID-19 was characterized by concentric left ventricular hypertrophy, right ventricular dilatation, severe hypokinesia and cardiac amyloidosis (19). Right ventricular dysfunction is a powerful indicator of the incidence and mortality of COVID-19-related heart injury (20, 21). In a systematic echocardiography study of COVID-19 patients with different ill severity, elevated troponin levels were associated with right ventricular dysfunction (22). Interestingly, clinical deterioration, including hemodynamic instability and a further increase in cardiac troponin, seems to rarely lead to left ventricular pump failure, and in most cases, it is accompanied by further dilatation of right ventricle and further deterioration of right ventricular function (22, 23).

COVID-19 patients with acute cardiac injury have a poor prognosis. The in-hospital complications of COVID-19 patients with acute myocardial injury increased significantly, and the clinical outcome deteriorated significantly (14, 16, 24). Complications such as acute kidney injury (8.5 vs. 0.3%), electrolyte imbalance (15.9 vs. 5.1%), and coagulation dysfunction (7.3 vs. 1.8%) were significantly higher in COVID-19 patients with heart injury (14). Life-threatening arrhythmias, including ventricular tachycardia and ventricular fibrillation (17.3 vs. 2%) were also significantly higher in COVID-19 patients with cardiac injury (16). More importantly, COVID-19-related acute myocardial injury was significantly associated with an increased mortality. Compared with patients without heart injury, patients with heart injury had a significantly higher mortality rate (51 vs. 4.5% and 59.6 vs. 8.9%) (14, 16). The increase of cardiac biomarkers may indicate a poor prognosis (16, 25, 26). In a study involving 1,099 COVID-19 patients from 552 hospitals, the expression of cardiac biomarkers was found to be significantly elevated in critically ill patients (7). Compared with mild non-ICU patients, CK-MB, LDH and hs-cTnI levels of severely ill patients admitted to ICU increased significantly (27). Guo et al. observed that the TnT and hsNT-proBNP levels significantly increased during hospitalization of deceased patients (16). The mortality of patients with elevated cardiac injury biomarkers was significantly higher than that of patients without cardiac injury (14). Therefore, the detection of cardiac biomarkers has certain clinical significance for judging the prognosis of COVID-19 hospitalized patients (28).

## Possible Pathological Mechanism Of Acute Myocardial Injury In Covid-19

Coronavirus is an enveloped, positive-stranded, single-stranded and highly diverse RNA virus family. The pathogenic and lethal SARS-CoV, Middle East respiratory syndrome coronavirus and SARS-CoV-2 all belong to this family (29). SARS-CoV-2 is a new type of β-coronavirus (large RNA virus) with a genome homology of up to 79.5% with SARS-CoV (30). The SARS-CoV-2 virus envelope is covered with spike S glycoprotein, which consists of two subunits S1 and S2. Subunit S1 has affinity for the ACE2 receptor on the cell surface, and subunit S2 fuses with the cell membrane. These two proteins work together to help the endocytosis of virus particles (31, 32). Different from SARS-CoV, the three-dimensional structure of the SARS-CoV-2 binding site is more compact, which not only improves the stability of the binding, but also enhances the binding affinity to the ACE2 receptor (33). In addition, SARS-CoV-2 contains a polyacid (furin) cleavage site inserted at the boundary of the S1/S2 subunits of the spike S protein (34). This furin binding site is unique and can enhance the ability of viruses to enter cells. It is a common feature of several highly pathogenic viruses including avian influenza.

**Figure 1:**
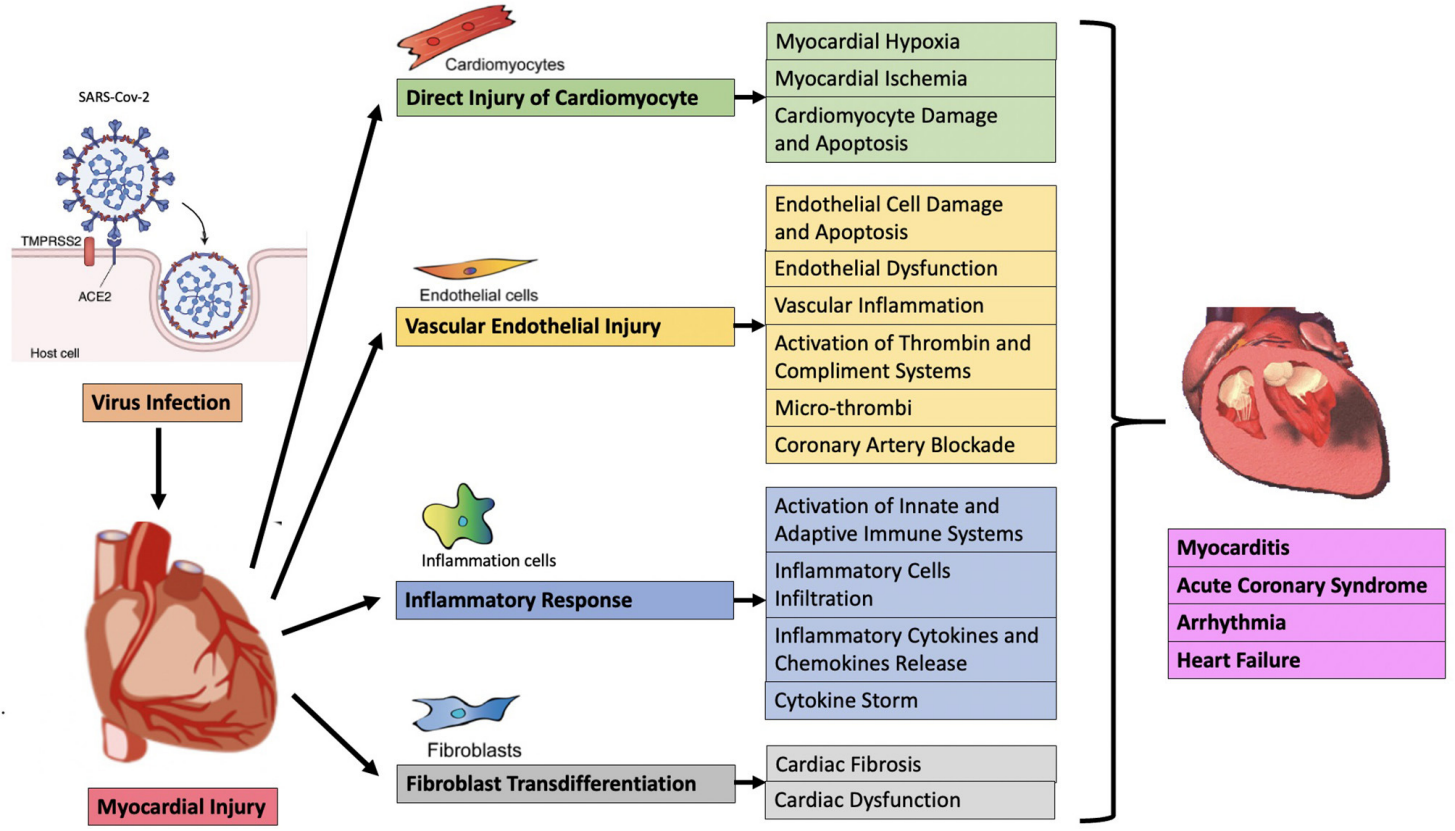
Possible pathological mechanism of acute myocardial injury in COVID-19.

At present, the mechanisms of acute myocardial injury in COVID-19 patients are unclear. Heart injury may be caused by direct or indirect mechanisms. The direct mechanism includes the virus invading the myocardial tissue, that is, direct infection, leading to the death and inflammation of myocardial cells. The indirect mechanism is secondary to respiratory failure and hypoxemia, leading to cardiac stress and hypoxia-related cardiomyocyte damage. Cardiac inflammation caused by severe systemic congestion is also included in the indirect mechanism (35), but it should be the third mechanism itself, because it may involve sepsis, toll-like receptor 4 (TLR4) activation and/or cytokine storm (also called “immune mediated cytokine release syndrome”) (36). In general, myocardial injury may be related to direct viral damage, inflammatory cell infiltration and pro-inflammatory cytokine release, oxidative stress, coronary vascular damage, endothelial damage with microthrombosis, myocardial interstitial fibrosis, and interferon-mediated immunity response, the excessive cytokine response of type 1 and type 2 helper T cells, the instability of coronary plaques and hypoxia (Figure 1) (37). Clarifying the underlying mechanisms of myocardial injury in COVID-19 are extremely important for finding therapeutic targets, reducing the severity of the disease, and reducing mortality.

### Angiotensin-Converting Enzyme-2 (ACE2) Receptor and Transmembrane Protease Serine 2 (TMPRSS2) Play a Key Role in COVID-19 Patients With Myocardial Injury

ACE2 is a type 1 transmembrane protein whose enzyme domain is located on the outer surface of the cell, where it can convert angiotensin II into angiotensin 1–7 (38). ACE2 is mainly expressed in the vascular endothelium of a variety of tissue structures, such as type I and type II lung cells, smooth muscle cells in the pulmonary vascular system, bronchial epithelium, epithelial cells in the lung, heart, intestine, blood vessels, testes and kidneys (39). ACE2 has an important immunomodulatory effect. On the one hand, ACE2 can directly interact with macrophages in the inflammatory environment of blood vessels and lungs to exert anti-inflammatory effects (40). On the other hand, ACE2 can reduce the pro-inflammatory and pro-oxidant effects of angiotensin II. Therefore, under pathological conditions, ACE2 has a positive effect on controlling excessive inflammation (41).

The main mechanism for SARS-CoV-2 to enter the host cell is through the ACE2 receptor (42). SARS-CoV-2 enters the lungs through the respiratory tract, and its spike S protein first binds to the ACE2 receptor on the surface of lung cells to mediate virus entry into the cell. On the one hand, the presence of TMPRSS2 can start the viral spike S protein to assist the virus to enter the cell and enhance the infectivity of the virus (43). On the other hand, after the virus binds to the ACE2 receptor and enters the cell, TMPRSS2 can help the virus to replicate in a large amount, so that the replicated virus further binds to other ACE2 receptors and promotes virus invasion. When the virus load in the lung overflows, it may attack multiple organs throughout the body, causing extensive tissue and organ damage.

ACE2 is widely expressed in the heart and lungs. It has been reported that compared with the lungs, the heart is the second major organ attacked by SARS-CoV-2 (44). Evaluation of the expression of ACE2 and TMPRSS2 by RNA sequencing of the hearts in normal people and COVID-19 patients found that ACE2 was most highly expressed in pericytes and was also clearly expressed in other cells such as cardiomyocytes, indicating that the heart may be an important target for SARS-CoV-2 infection (45, 46). The expression of ACE2 in the heart of COVID-19 patients is significantly increased, and direct cardiac injury is prone to occur (44, 47). Studies found that TLR4 played an important role in the pathogenesis of SARS-CoV-2. SARS-CoV-2 may bind and activate TLR4 to increase the expression of ACE2, promote virus entry and cause excessive or long-term inflammation (48). Once SARS-CoV-2 targets ACE2, it may increase the level of angiotensin II through the action of NADPH oxidase activity, and may cause endothelial dysfunction and chronic myocardial hypoxia (49).

The internalization of the virus and ACE2 causes the loss of ACE2 on the cell surface, leading to increased levels of angiotensin II and decreased levels of angiotensin 1-7. Sakamoto et al. (50) performed ACE2 and TMPRSS2 immunostaining on the hearts of 15 COVID-19 patients from Bergamo, Italy. They observed that ACE2 was mainly located in the cell membrane, TMPRSS2 was mainly located in the nucleus and cytoplasm, and ACE2 and TMPRSS2 were extremely low in the heart of COVID-19 patients (50). There was also evidence in preclinical models using SARS-CoV-2 that due to the binding of the virus to the ACE2 receptor during infection, ACE2 in the heart was significantly down-regulated (51). Oudit et al. found that the expression of ACE2 in the myocardium of mice infected with SARS-CoV-2 was significantly reduced, and SARS-CoV-2 could reduce the expression of ACE2 due to internalization (51). The reduction of ACE2 expression has a dual effect. On the one hand, the host can defend against infection and limit the continuous proliferation of the virus. On the other hand, the biological effect of ACE2 is also significantly weakened, and it cannot limit the pro-inflammatory, pro-thrombotic and pro-oxidant effects of angiotensin II, the cardioprotective effect is weakened, and the risk of heart failure increases. Although this virus proliferates at a low level in the heart of the host, it may cause potential danger signals to be further released to the immune system, triggering an excessive downstream inflammatory response. When there is a slight increase in troponin, it indicates the appearance of virus or immune-mediated myocardial injury. If the immune response continues to increase, it will further aggravate the heart injury. When the heart biomarkers continue to increase, it often indicates an inflammatory storm and a poor prognosis. Hydroxychloroquine has been confirmed to bind cell surface sialic acid and gangliosides with high affinity, thereby impairing SARS-CoV-2 spike protein recognition and binding to host cell ACE2 receptors (52).

### The Direct Injury of SARS-CoV-2 to Heart in COVID-19

Previous literature analysis of the hearts of patients who died of SARS showed that SARS-CoV virus RNA was detected in 35% of the hearts (51). After SARS-CoV-2 infects human body, combining with ACE2 on the surface of cardiac epithelial cells directly damages myocardium and causes its dysfunction is a possible damage mechanism. The high expression of ACE2 in cardiomyocytes, pericytes, fibroblasts, endothelial cells, epicardial adipocytes and smooth muscle cells supports the mechanism of direct viral damage (39, 53). After the virus invades the heart, it can induce inflammatory cells to infiltrate the pericardium, myocardium and intima. Immune cells such as neutrophils, pro-inflammatory monocytes/macrophages and lymphocytes gather around the area of the myocardium infiltrated by the virus. Many cases reported myocarditis induced by COVID-19. These findings support the hypothesis that SARS-CoV-2 directly damages the myocardium (13, 54, 55). Lindner et al. studied the heart tissues of 39 patients whose throat swabs tested positive for SARS-CoV-2 through autopsy and found that SARS-CoV-2 virus was present in the hearts of 24 cases (61.5%), of which 16 cases (41 %) the viral load copy number was greater than 1000 copies/μg RNA (56). However, there are few studies to detect the virus carried by cardiomyocytes. Sakamoto et al. used real-time polymerase chain reaction to detect the expression of SARS-CoV-2 in the hearts of 15 patients with COVID-19, and found that the virus was only present in the left atrium of one patient, and there was no virus in any of the heart chambers of the remaining patients. In addition, *in-situ* hybridization (ISH) and indirect immunofluorescence showed that SARS-CoV-2 was localized in the myocytes in the left atrium (50). Electron microscopy detected virus particles in the cardiac myocytes, endothelial cells and fibroblasts of an 11-year-old boy who developed multiple system inflammatory syndrome and died of heart failure (57). In a patient with a rapid onset of myocardial injury caused by SARS-CoV-2 infection, myocardial biopsy showed that there were virus particles in the interstitial tissue, but there were no virus particles in the cardiomyocytes (58). Nevertheless, the direct invasion of the myocardium by the virus is still a possible damage mechanism. Antiviral therapy can effectively inhibit the invasion of SARS-CoV-2, including chloroquine and hydroxychloroquine, type 1 interferons (IFN–I), lopinavir/ritonavir (Kaletra) and remdesivir (59).

### Activation of Immune System and Imbalance of Immune Response in COVID-19 With Myocardial Injury

The human immune system is a multi-stage network that protects the body from harmful bacteria, viruses, and other organisms. The occurrence and development of COVID-19 is mediated by viruses and individual immune systems. To successfully defend against viral infection, the body relies on innate and adaptive immune systems. The innate immune system is the first line of defense and is responsible for identifying certain structurally conserved components of the virus: early production of interferons, inflammatory cytokines and chemokines. Interferons and other cytokines recruit immune cells (such as natural killer cells, neutrophils, and monocytes) for subsequent cytotoxic and adaptive immune responses. Specific B lymphocytes and T lymphocytes are part of the adaptive immune system. Their role is to act as a barrier to delay the replication of the virus, to produce specific neutralizing antibodies against the virus to avoid further infection, and to form a persistent memory against the virus (60). However, when the infected cells proliferate beyond the range of T cells, the patient's condition may rapidly deteriorate. The endothelium plays a role in controlling the immune system through various receptors and cytoplasmic proteins (61). Endothelial cells secrete cytokines to regulate innate and adaptive immune responses, and recruit immune cells at their sites of action (62). Endothelial activation increases the permeability of blood vessels to plasma proteins, releases pro-inflammatory cytokines, tumor necrosis factor-α (TNF-α), chemokines, adhesion molecules (from activated white blood cells) and induces inflammation (63). Endothelial dysfunction stimulates a series of signaling molecules to release nuclear factor-κB (NF-κB) to limit the innate and adaptive immune response (64). If the innate immunity cannot eliminate the threat, it will activate the adaptive immune response, turning acute inflammation into chronic inflammation (65). The innate immune response (such as increased neutrophils, pro-inflammatory macrophages, and lymphopenia) and adaptive immune responses (activation of CD4^+^ T cells and CD8^+^ T cells) play important roles in autoimmunity or anti-inflammation of COVID-19 patients (66). When the immune regulation dysfunction, the human body cannot cope with the virus infection and enters a critical stage, and a strong and harmful inflammatory reaction occurs.

### Systemic Inflammatory Response Syndrome and Cytokine Storm Leads to Myocardial Injury in COVID-19

Excessive systemic inflammation can occur after infected with COVID-19, including cytokine storm-like results with extremely high mortality. Such COVID-19 patients with a strong immune response may present with acute myocarditis, heart failure or cardiogenic shock, accompanied by hypercytokineemia and cardiac inflammatory cell infiltration (67). Studies showed that leukocytosis (especially neutrophilia) was a high inflammatory response to SARS-CoV-2 infection and/or secondary bacterial infection, which can aggravate the severity of the disease and promote myocardial injury (68). In addition, more than 80% of patients had lymphopenia after infected with SARS-CoV-2. The degree of lymphopenia was a very important prognostic indicator in the early stage of infection. In the early analysis of patients who died of COVID-19, the most notable findings were the significant decrease in circulating CD4^+^ and CD8^+^ T lymphocyte levels, and monocytes (monocytes and macrophages) were relatively dominant in the damaged myocardium (69, 70).

With the consumption of CD4^+^ T cells, the decrease of regulatory T cell function, the decrease of progressive lymphocytes, and the aging immune system lead to the proliferation of steady-state lymphocytes, which has the characteristics of autoimmunity and excessive inflammation (71). The ability of senescent macrophages to swallow apoptotic cells decreases, leading to a general pro-inflammatory state. When the elderly population is infected with SARS-CoV-2, the unbalanced immune system of the elderly will worsen, which will further aggravate the exhaustion of CD4^+^ T cells and the inflammatory macrophage response, and expand the systemic inflammatory response. This is also one of the reasons why the incidence of critical illness and mortality in the elderly are more frequent after being infected with COVID-19. In addition, epicardial adipose tissue may be directly related to myocardial injury. It is a high inflammatory response reservoir with a large number of macrophage infiltration and rich pro-inflammatory cytokines. This may be the reason why obese patients have higher mortality and complication rates (72).

The autopsy results of the myocardial tissue of patients who died from COVID-19 support the hypothesis that systemic inflammation may be the driving factor of heart damage. Analysis of myocardial tissue showed that there was a small amount of inflammatory infiltration of monocytes in the myocardial interstitium without substantial myocardial damage (73). In addition, a study of 112 hospitalized COVID-19 patients also showed that the cardiac injury was attributed to systemic cytokines rather than the direct damage to the heart by the virus (26).

Another main hypothetical mechanism of myocardial injury may be the release of various pro-inflammatory cytokines during inflammation, such as interleukin-1 (IL-1), beta interferon-gamma (IFN-γ), macrophage inflammatory protein (MIP)-1A, TNF-α as well as IL-6 (74). SARS-CoV-2 mainly invades the lungs through ACE2 receptors and progresses to pneumonia and acute respiratory distress syndrome (ARDS). According to the viral load, the infection can be further spread through the ACE2 receptor to various organs, such as the heart, liver, kidney, brain, endothelium, gastrointestinal tract, immune cells. When the virus invades the human body and damages the organs, systemic pro-inflammatory factors are activated, the inflammation self-amplifies and triggers an inflammatory storm, leading to systemic inflammation and toxicity. Mcp-1 is one of the significantly increased cytokines, and it is also the main regulator of the migration and infiltration of the monocyte/macrophage system to the SARS-CoV-2 infection site. IL-1β is a key regulator of inflammatory response, which can stimulate the release of other cytokines (such as IL-17 and IL-21), and can also increase cell proliferation and differentiation (75). Studies showed that the inflammatory response level of IL-1β elevated in COVID-19 patients, especially in patients with poor prognosis. Subsequently, on the basis of the increase in IL-1β, the level of IL-6 also increased, which may herald the upcoming cytokine storm. Cytokine storm will further aggravate myocardial injury, and the widespread and malignant combination of cytokine and organ crosstalk leads to systemic excessive inflammation and ultimately to cardiac dysfunction (35). In addition, the continuous increase of inflammatory cytokines after COVID-19 infection may eventually lead to the reduction of coronary blood flow, oxygen supply, microthrombosis and the degeneration of coronary plaques (16). Anti-inflammatory agents, such as glucocorticoids and Tocilizumab, a recombinant-humanized monoclonal antibody targeting both soluble and membrane-bound forms of IL-6 receptor, could effectively reduce the release of inflammatory factors (59). Besides, as a potential antithrombotic drug, although the overall beneficial effect of chloroquine is not clear in COVID-19, it has been confirmed can reduce neutrophil extracellular trap formation, platelet aggregation and circulating tissue factor in mice (52).

### Vascular Endothelial Injury, Microthrombosis and Myocardial Ischemia Are Associated With Myocardial Injury in COVID-19

Another potential mechanism of cardiac injury is that the virus directly enters the heart endothelial cells instead of the cardiomyocytes. There are also ACE2 receptors on the surface of endothelial cells. After SARS-CoV-2 invades these epithelial cell membranes, it damages the endothelium, triggers the overproduction of thrombin, inhibits fibrinolysis, activates the complement pathway, triggers thrombus inflammation, and ultimately leads to microthrombus deposition and microvascular dysfunction (76, 77). Through electron microscopy and virus particle identification, evidence of direct endothelial infection has been recorded in cardiac endothelial cells at autopsy (76, 78). Excessive immune system activation, activation of inflammatory cells (such as macrophages) and the release of inflammatory mediators (such as IL-1β, IL-6, TNF-α) can further promote endothelial cell activation and release more pro-inflammatory cytokines, promote the expression of adhesion molecules (such as ICAM-1, VCAM-1), and recruit more leukocyte infiltration and platelet activation and aggregation. In addition, circulating cytokines can stimulate the expression of macrophages and leukocyte adhesion molecules on endothelial cells with potential atherosclerotic lesions, making them more susceptible to destruction and increasing the possibility of clinically obvious acute coronary syndromes (79, 80). Systemic cytokines may also activate microvascular endothelial cells, leading to coronary microvascular dysfunction, myocardial ischemia and myocardial damage (79). The inflammation and subsequent dysfunction of endothelial cells in the heart are the result of the direct effect of SARS-CoV-2 infection of endothelial cells and the indirect effect of host inflammatory response. Dysfunctional endothelial cells become adhesion promoters and coagulants, further accelerating vascular inflammation, enhancing the prethrombotic state, increasing the levels of D-dimers and fibrin degradation products (FDPs), and even appearing microthrombus (81). The formation of microthrombus can make patients prone to cardiac micro-infarction, further aggravating the state of myocardial injury and heart failure. Changes in the coagulation and fibrinolytic system are important in COVID-19 patients, and diffuse intravascular coagulation (DIC) has been observed in most patients who died (82). Microthrombosis caused by damaged endothelium or hypercoagulable state can make existing coronary plaques unstable, leading to type I myocardial ischemia (83). The right ventricle is the most prone to ischemia and dysfunction. This is due to the sudden increase of the right ventricular afterload caused by microthrombosis and the mismatch between the supply and demand of the coronary arteries. In addition, endothelial and microvascular damage in COVID-19 leads to increased inflammation, increased coronary capillary permeability, vasospasm, decreased myocardial perfusion and myocardial ischemia, and increased oxygen supply/demand leading to vascular homeostasis damage are all incentives for right ventricular dysfunction (84). More than 50% of patients with right ventricular dysfunction are associated with moderate to severe ARDS and are a recognized determinant of mortality (84). In addition to coagulation disorders, endothelial damage may also lead to increased vascular permeability and decreased levels of nitric oxide in the lining of capillaries (85). All these factors can cause severe cardiac injury and eventually heart failure.

### Myocardial Injury Related to Myocardial Hypoxia in COVID-19

SARS-CoV-2 infection may be related to myocardial injury by increasing myocardial oxygen demand and reducing myocardial oxygen supply. Myocardial injury is secondary to the imbalance of myocardial oxygen supply and demand without rupture of atherosclerotic plaque, which is called type 2 myocardial infarction. Hypoxia caused by severe respiratory-related complications (such as ARDS/respiratory failure) is common in myocardial injury in COVID-19 patients (4, 7, 86). Hypoxia caused by respiratory failure may put an additional burden on the heart, and is associated with increased biomarkers of myocardial injury, leading to a worse prognosis (4). Pulmonary vasculitis with extensive vascular thrombosis, microangiopathy and alveolar capillary occlusion, combined with the overall prethrombotic state, can promote pulmonary embolism and lead to hypoxemia (87, 88). Increased myocardial oxygen demand in severe COVID-19 patients secondary to tachycardia, increased cardiac output, and increased right ventricular afterload. Decreased oxygen supply will reduce the hydrolysis of adenosine triphosphate (ATP), significantly reduce the energy supply for cell metabolism, increase anaerobic fermentation, and cause intracellular acidosis. Oxygen free radicals damage the phospholipid layer of the cell membrane, leading to cell membrane damage and mitochondrial damage, thereby further reducing ATP synthesis. The imbalance between increased oxygen demand and decreased supply is likely to cause subendocardial ischemia. At the same time, the influx of calcium ions induced by hypoxia also lead to cardiomyocyte damage and apoptosis (12). Hypotension is a common clinical feature of sepsis and cytokine storm syndrome, and can also reduce myocardial oxygen supply (79). In addition, systemic infection and fever increase the metabolic demands of peripheral tissues and end-organs, thereby increasing the metabolic demands of cardiomyocytes (89).

### Other Potential Mechanisms of Myocardial Injury in COVID-19

Myocardial interstitial fibrosis may be another mode of myocardial injury. The infiltration of neutrophils, macrophages and CD4^+^ T lymphocytes in patients with COVID-19 can promote the activation of fibroblasts to myocardial fibroblasts, causing pathological heart remodeling and fibrosis, leading to the development of heart failure and early death of infected patients. Zhao et al. found that SARS virus activated TGF-β signaling through the Smad pathway to induce pulmonary fibrosis, which is also a common pathway for myocardial interstitial fibrosis (90). It was also suggested that in COVID-19 patients, a strong interferon-mediated response may contribute to myocardial function. Specifically, it is mainly the transition of interferon from overactive innate immunity to protective adaptive immunity (91, 92). Secondly, pro-inflammatory cytokines can promote the formation of reactive oxygen species and free radicals, cause oxidative stress, and ultimately lead to the depletion of NAD^+^ and ATP, resulting in apoptosis and necrosis of cardiomyocytes. Finally, another proposed mechanism is the overreaction of type 1 and type 2 helper T cells to cytokines (93). The above are all possible mechanisms of myocardial injury, but the specific targets and pathways of action need to be further verified.

## Conclusion

Myocardial injury in COVID-19 is usually accompanied by abnormal electrocardiogram and echocardiography, and increased inflammation markers. The mechanisms of myocardial injury in COVID-19 maybe include direct injury of myocardial cells, systemic inflammatory response, hypoxia, prethrombotic and procoagulant state, myocardial interstitial fibrosis, interferon-mediated immune response, coronary artery plaque instability and other related factors. And angiotensin-converting enzyme-2 receptor may play a key role in the myocardial injury in COVID-19. A correct understanding of COVID-19's damage to the heart is of positive significance for early recognition, rapid diagnosis, and drug intervention, and has an important impact on reducing the incidence and mortality of myocardial injury in COVID-19 patients.

## Author Contributions

SL completed and revised the manuscript. JW searched literature and completed partial writing. WG reviewed and revised the article. YY and ZZ searched literature. SN revised the article. All authors contributed to the article and approved the submitted version.

## Funding

This study was funded by grants from Beijing Natural Science Foundation (7191002), the National Natural Science Foundation of China (81970292, 81600213, 81670222, and 81700262), and Beijing Hospitals Authority Youth Program (QML20190603).

## Conflict of Interest

The authors declare that the research was conducted in the absence of any commercial or financial relationships that could be construed as a potential conflict of interest.

## Publisher's Note

All claims expressed in this article are solely those of the authors and do not necessarily represent those of their affiliated organizations, or those of the publisher, the editors and the reviewers. Any product that may be evaluated in this article, or claim that may be made by its manufacturer, is not guaranteed or endorsed by the publisher.
